# Supplementary material for: Disaggregating Asian American and Pacific Islander Risk of Fatal Police Violence
Source: PLoS One. 2022 Oct 10;17(10):e0274745. doi: 10.1371/journal.pone.0274745 (PMC9550032; doi:10.1371/journal.pone.0274745)
Supplement: S1 Appendix — (DOCX) [file pone.0274745.s001.docx]

**Appendix to “Disaggregating Asian American and Pacific Islander risk of fatal police violence”**

This appendix is divided into five sections, providing details on our data, methodology, and results:

1. Generating our list of AAPI decedents
2. Identifying decedents’ national/ethnic and regional backgrounds
3. Regional background classifications
4. Characteristics of decedents
5. Point estimates and confidence intervals by regional, national/ethnic, and racial background

*Appendix Tables & Figures:*

**S1 Fig.** Identification Flowchart

**S1 Table.** Classification Method for Each Ethnic Group

**S2 Fig.** Proportion of agreement between NamePrism and all hand-codes with a matching probability of at least X or higher

**S2 Table.** AAPI region-country/ethnicity crosswalk

**S3 Table.** Characteristics of decedents by regional and national/ethnic background

**S4 Table.** Estimates of the annual rate of fatal police violence for different racial/ethnic groups

**1. Generating our list of AAPI decedents**

We first downloaded the entire Fatal Encounters database on 10/7/2020, which is publicly available online at no charge. Fatal Encounters started tracking police-involved fatalities in real time in 2013; it also began retrospective searches for these fatalities going back as far as 2000. Because widespread media coverage of these deaths was less common before the early 2010s (when the Black Lives Matter movement came to prominence), the accuracy of retrospective record-keeping and race classification within Fatal Encounters is likely to be lower before 2013. We thus subset to fatalities occurring in 2013 through 2019, the last complete year of data available at the time of our download. We further excluded deaths whose causes could be considered “accidents,” retaining only those decedents who were lethally shot, tasered, asphyxiated, beaten, or whose cause of death was unknown.

To get our list of AAPI decedents, we then further subset to those listed as “Asian/Pacific Islander” under Fatal Encounters’ “race” variable; we did not rely on FE’s imputed race fields. This provided an initial list for hand-coding. To ensure South Asian people were not incorrectly identified as Middle Eastern, we also hand-coded those listed as “Middle Eastern” in Fatal Encounters, adding to our list decedents who we could verify as South Asian and coding the remainder as White, given that many people of “Middle Eastern” descent are listed as White on the Census (the source of our denominator data).

To ensure we were not missing decedents and reduce the risk of race misclassification, we cross-checked our list from Fatal Encounters with Mapping Police Violence, a similar citizen science initiative that maintains a database of police killings. MPV classifies decedents’ race/ethnicity by searching social media, obituaries, criminal records databases, and police reports. We downloaded a list of all AAPI decedents in MPV and checked it against our list from Fatal Encounters. In cases of disagreement between MPV and FE, we first attempted to adjudicate with hand-coding. If we could not corroborate MPV’s classifications, we defaulted to FE’s.

If our web searches yielded documentation that decedents listed as AAPI in FE were in fact of other racial/ethnic backgrounds, those decedents were removed.

**2. Identifying decedents’ national/ethnic and regional backgrounds**

We identified AAPI decedents in four steps, visualized in Figure S1: (1) “hand-coding” via web searches, (2) pulling in “Pacific Islander” racial classifications from Mapping Police Violence (MPV), (3) using a surname list developed by Lauderdale et al. [1], and (4) using NamePrism, a classification algorithm developed by Ye and colleagues [2].

*S1 Figure. Identification Flowchart*

In the first step, we conducted web searches that included various combinations of the names of decedents and the words “police,” “shot” or “killed,” as well as the locations and dates at which decedents were killed, if necessary. We followed related links and documentation provided by news sources, in official records, or in obituaries.

In the second step, we used MPV’s racial classifications to identify decedents’ backgrounds as Pacific Islander. MPV treats Asian and Pacific Islander as separate categories; in cases where no hand-codes were available, we used MPV to identify AAPI decedents as Pacific Islanders.

In the third step, we imputed national/ethnic background using a list generated by Lauderdale [1]. This list included only Chinese, Filipino, Japanese, Vietnamese, Korean, or Indian surnames. Among hand-coded cases, there was 66% agreement with the Lauderdale imputed ethnicity for these ethnic categories, suggesting reasonable fidelity.

In the fourth step, we imputed national/ethnic background using NamePrism, a name classification tool developed by Ye et al. that used word embedding methods to generate a taxonomy of 39 leaf nationalities in a training set of 57 million contact lists in 118 countries from an email company. Name Prism provides a predicted ethnic classification with a probability of accuracy. We only used Name Prism’s ethnicity classification for those with a probability of at least 50% (see Figure S2). Using this cutoff, Name Prism correctly identified 84% of hand-coded national/ethnic backgrounds and 92% of hand-coded regional backgrounds.

*S1 Table. Classification Method for Each Ethnic Group*

|  | Hand Coded | Lauderdale | Name Prism | Missing |
| --- | --- | --- | --- | --- |
| Bangladesh | 1 (100.00%) | 0 (0.00%) | 0 (0.00%) | 0 (0.00%) |
| Burma | 1 (100.00%) | 0 (0.00%) | 0 (0.00%) | 0 (0.00%) |
| Cambodia | 5 (100.00%) | 0 (0.00%) | 0 (0.00%) | 0 (0.00%) |
| China | 7 (43.75%) | 7 (43.75%) | 2 (12.50%) | 0 (0.00%) |
| Fiji | 1 (100.00%) | 0 (0.00%) | 0 (0.00%) | 0 (0.00%) |
| Guam | 1 (100.00%) | 0 (0.00%) | 0 (0.00%) | 0 (0.00%) |
| Hawaii | 2 (100.00%) | 0 (0.00%) | 0 (0.00%) | 0 (0.00%) |
| Hmong | 5 (100.00%) | 0 (0.00%) | 0 (0.00%) | 0 (0.00%) |
| India | 4 (57.14%) | 3 (42.86%) | 0 (0.00%) | 0 (0.00%) |
| Japan | 0 (0.00%) | 1 (100.00%) | 0 (0.00%) | 0 (0.00%) |
| Korea | 3 (75.00%) | 1 (25.00%) | 0 (0.00%) | 0 (0.00%) |
| Laos | 5 (100.00%) | 0 (0.00%) | 0 (0.00%) | 0 (0.00%) |
| Malaysia | 1 (100.00%) | 0 (0.00%) | 0 (0.00%) | 0 (0.00%) |
| Marshall Islands | 1 (100.00%) | 0 (0.00%) | 0 (0.00%) | 0 (0.00%) |
| Micronesia | 3 (100.00%) | 0 (0.00%) | 0 (0.00%) | 0 (0.00%) |
| Nepal | 1 (100.00%) | 0 (0.00%) | 0 (0.00%) | 0 (0.00%) |
| Pakistan | 4 (100.00%) | 0 (0.00%) | 0 (0.00%) | 0 (0.00%) |
| Philippines | 12 (70.59%) | 4 (23.53%) | 1 (5.88%) | 0 (0.00%) |
| Samoa | 4 (100.00%) | 0 (0.00%) | 0 (0.00%) | 0 (0.00%) |
| Sri Lanka | 1 (100.00%) | 0 (0.00%) | 0 (0.00%) | 0 (0.00%) |
| Thailand | 2 (100.00%) | 0 (0.00%) | 0 (0.00%) | 0 (0.00%) |
| Tonga | 1 (100.00%) | 0 (0.00%) | 0 (0.00%) | 0 (0.00%) |
| Vietnam | 16 (57.14%) | 10 (35.71%) | 2 (7.14%) | 0 (0.00%) |
| Missing | 0 (0.00%) | 0 (0.00%) | 0 (0.00%) | 31 (100.00%) |

*S2 Figure. Proportion of agreement between NamePrism and all hand-codes with a matching probability of at least X or higher*

1. *National/ethnic background agreement*


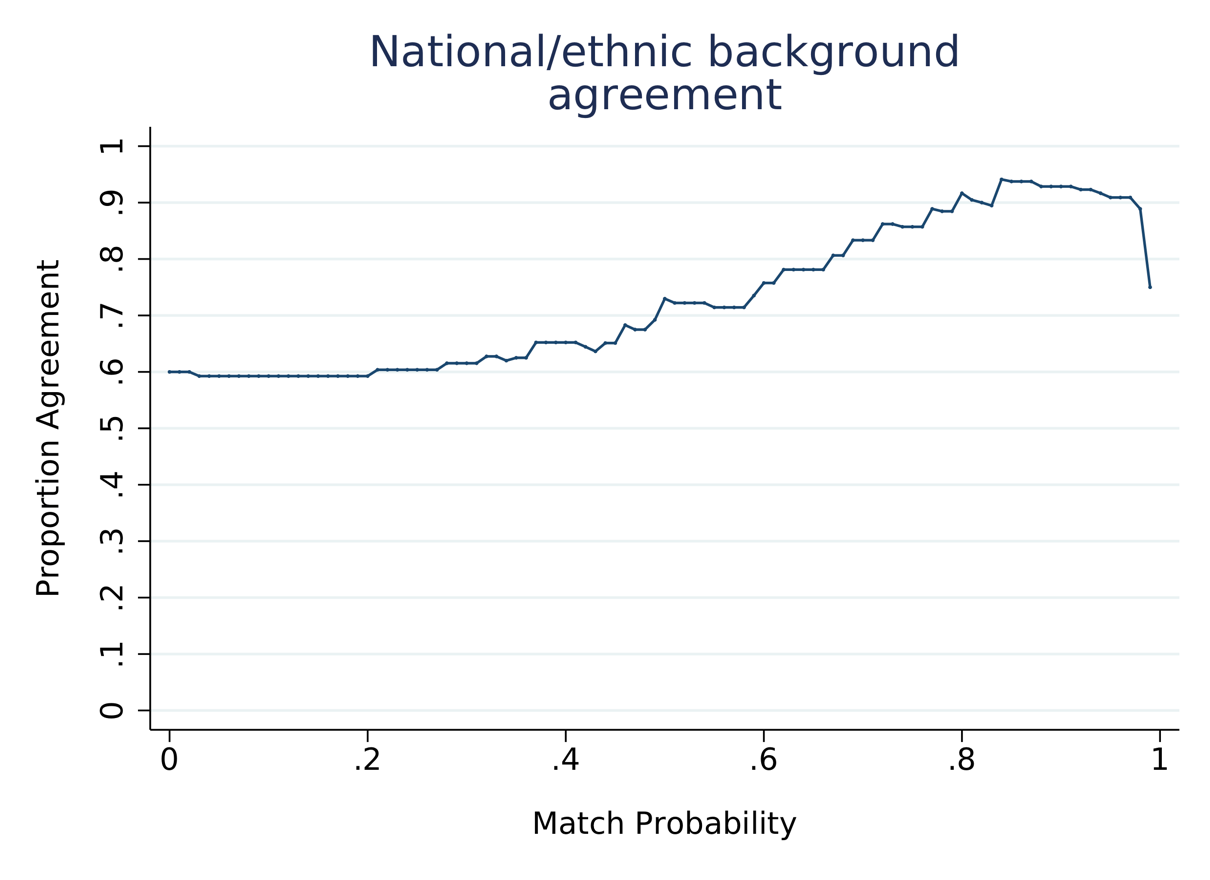


1. *Regional background agreement*


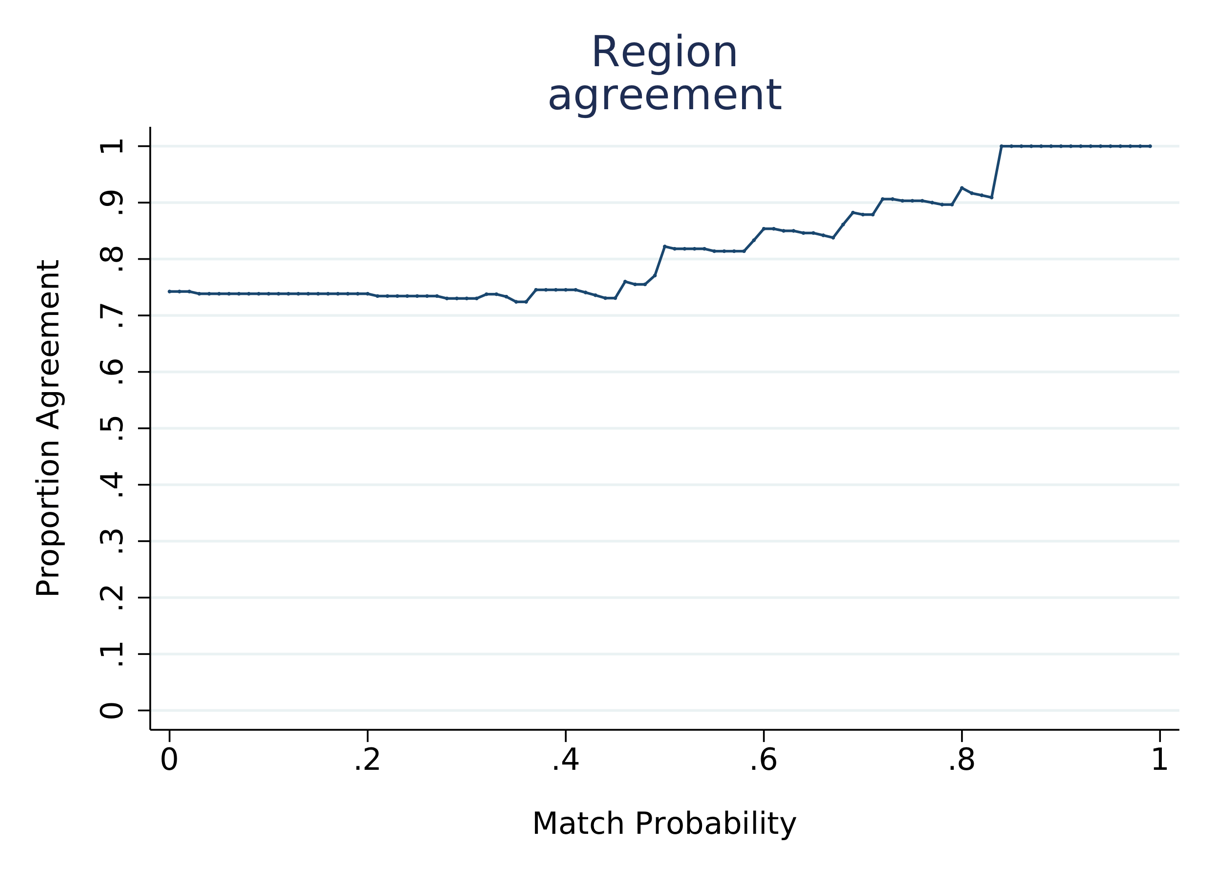


**3. Regional background classifications**

Within Asia, we assigned national/ethnic backgrounds to larger regions. Not all of these national/ethnic backgrounds had corresponding, disaggregated denominator data in the US Census. A crosswalk is provided in Table S2.

*S2 Table. AAPI region-country/ethnicity crosswalk*

| **National/Ethnic Background** | **Region** | **Census Denominator Available** |
| --- | --- | --- |
| China | East | 1 |
| Japan | East | 1 |
| Mongolia | East | 1 |
| North Korea | East | 1 |
| South Korea | East | 1 |
| Taiwan | East | 1 |
| Bangladesh | South | 1 |
| Bhutan | South | 1 |
| India | South | 1 |
| Maldives | South | 0 |
| Nepal | South | 1 |
| Pakistan | South | 1 |
| Sri Lanka | South | 1 |
| Brunei | Southeast | 0 |
| Cambodia | Southeast | 1 |
| Hmong | Southeast | 1 |
| Indonesia | Southeast | 1 |
| Laos | Southeast | 1 |
| Malaysia | Southeast | 1 |
| Myanmar | Southeast | 0 |
| Philippines | Southeast | 1 |
| Singapore | Southeast | 0 |
| Thailand | Southeast | 1 |
| Timor-Leste | Southeast | 0 |
| Vietnam | Southeast | 1 |

**4. Characteristics of decedents**

*S3 Table. Characteristics of decedents by regional and national/ethnic background*

**
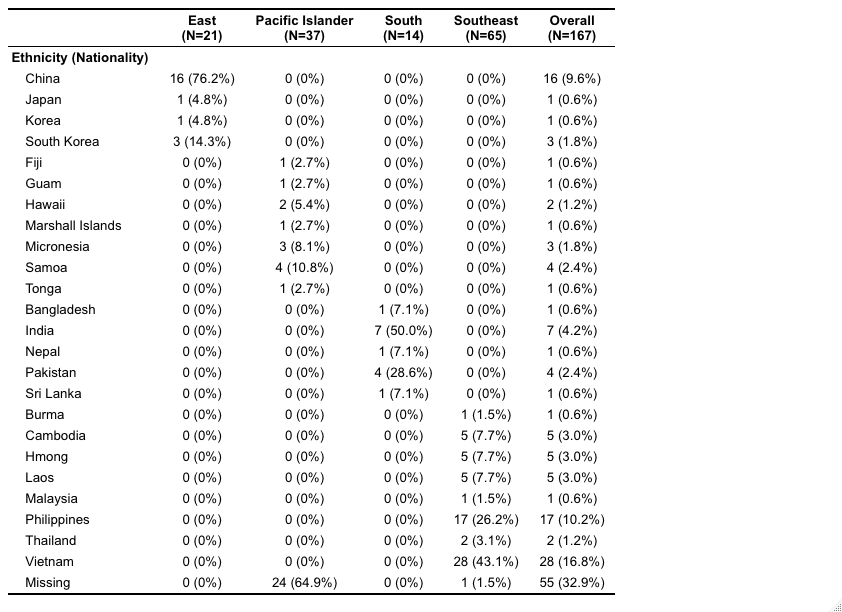
**

**5. Point estimates and confidence intervals by regional, national/ethnic, and racial background**

Point estimates by racial/ethnic, AAPI regional, and AAPI national/ethnic background are presented below. These estimates are visualized in the main text in Figures 1 and 2. Not all national/ethnic groups had corresponding, disaggregated denominators in the US Census; rates for these groups could not be calculated. Please contact authors for our full data, including the national/ethnic classifications of each of the 167 AAPI decedents in our analysis.

*S4 Table. Estimates of the annual rate of fatal police violence for different racial/ethnic groups*

| **Racial/Ethnic Group** | **Estimate** | **SE** | **95% CI** |
| --- | --- | --- | --- |
| *By traditional racial/ethnic group* | |  |  |
| AIAN | 0.89 | 0.13 | (0.63 ,1.14) |
| API | 0.13 | 0.01 | (0.11 ,0.15) |
| Black | 0.79 | 0.01 | (0.76 ,0.82) |
| Hispanic | 0.35 | 0.01 | (0.34 ,0.37) |
| White | 0.30 | 0.01 | (0.28 ,0.32) |
| *By AAPI regional background* | |  |  |
| East | 0.05 | 0.00 | (0.04, 0.06) |
| Pacific Islander | 0.88 | 0.14 | (0.61, 1.15) |
| South | 0.04 | 0.01 | (0.02, 0.07) |
| Southeast | 0.16 | 0.01 | (0.13, 0.19) |
| *By AAPI national/ethnic background* | | |  |
| Bangladesh | 0.08 | 0.08 | (-0.07, 0.24) |
| Bhutan | 0.00 | 0.00 | (0.00, 0.00) |
| Myanmar | 0.09 | 0.08 | (-0.07, 0.25) |
| Cambodia | 0.27 | 0.15 | (-0.02, 0.57) |
| China | 0.06 | 0.01 | (0.04, 0.07) |
| Fiji | 0.40 | 0.37 | (-0.33, 1.12) |
| Guam | 0.17 | 0.15 | (-0.14, 0.47) |
| Hawaii | 0.16 | 0.09 | (-0.03, 0.34) |
| Hmong | 0.25 | 0.09 | (0.07, 0.43) |
| India | 0.03 | 0.01 | (0.01, 0.05) |
| Indonesia | 0.00 | 0.00 | (0.00, 0.00) |
| Japan | 0.02 | 0.02 | (-0.02, 0.05) |
| Korea | 0.01 | 0.01 | (-0.01, 0.03) |
| Laos | 0.35 | 0.19 | (-0.02, 0.72) |
| Malaysia | 0.72 | 0.67 | (-0.59, 2.04) |
| Marshall Islands | 0.48 | 0.45 | (-0.39, 1.36) |
| Mongolia | 0.00 | 0.00 | (0.00, 0.00) |
| Nepal | 0.09 | 0.09 | (-0.08, 0.27) |
| Pakistan | 0.00 | 0.00 | (0.00, 0.00) |
| Philippines | 0.09 | 0.02 | (0.04, 0.13) |
| Samoa | 0.50 | 0.24 | (0.03, 0.97) |
| Sri Lanka | 0.29 | 0.27 | (-0.24, 0.82) |
| Taiwan | 0.00 | 0.00 | (0.00, 0.00) |
| Thailand | 0.14 | 0.09 | (-0.02, 0.31) |
| Tonga | 0.34 | 0.31 | (-0.28, 0.95) |
| Vietnam | 0.22 | 0.04 | (0.15, 0.30) |

**References**

1 Lauderdale DS, Kestenbaum B. Asian American ethnic identification by surname. *Popul Res Policy Rev* 2000;**19**:283–300. doi:10.1023/A:1026582308352

2 Ye J, Han S, Hu Y, *et al.* Nationality Classification Using Name Embeddings. *Int Conf Inf Knowl Manag Proc* 2017;**Part F1318**:1897–906.http://arxiv.org/abs/1708.07903 (accessed 7 Apr 2021).
